# A systematic review and dose-response meta-analysis of red blood cell distribution width to albumin ratio as mortality predictor in cardiovascular disease

**DOI:** 10.1038/s41598-024-81876-z

**Published:** 2025-09-01

**Authors:** Ihsan Fachry Arba, Chaq El Chaq Zamzam Multazam, Wynne Widiarti, Paulus Parholong Siahaan, Yudi Her Oktaviono, David Setyobudi, Pandit Bagus Tri Saputra, Ariikah Dyah Lamara, Jannatin Nisa’ Arnindita

**Affiliations:** 1 Faculty of Medicine, Universitas Airlangga, Surabaya, Indonesia; 2 National Heart and Lung Institute, Imperial College London, London, UK; 3 Department of Cardiology and Vascular Medicine, Faculty of Medicine, Universitas Airlangga – Dr. Soetomo General Academic Hospital, Jl. Mayjen Prof. Dr. Moestopo No 6–8, Surabaya, East Java, 60285 Indonesia; 4 Cardiovascular Research and Innovation Center, Universitas Airlangga, Surabaya, Indonesia

**Keywords:** Red blood cell distribution width-to-albumin ratio, Cardiovascular disease, Dose-response meta-analysis, Prognostic biomarker, Mortality, Prognostic markers, Cardiovascular diseases

## Abstract

Red blood cell distribution width (RDW) and albumin separately have been used as mortality predictors for people with cardiovascular disease (CVD). This study aims to explore whether the RDW-to-albumin ratio (RAR) could provide a better prognostication in the CVD population. A systematic search of suitable studies was conducted in PubMed, Web of Science, Scopus, and ProQuest until February 1, 2024. Mortality and length of stay outcomes of the highest vs. lowest RAR tertile were pooled using hazard ratio (HR) and standardized mean difference (SMD), respectively. Additionally, a dose-response meta-analysis was performed. Publication bias, subgroup, and sensitivity analyses were conducted to address the causes of heterogeneity. Sixteen studies with 30,933 participants were included in the meta-analysis. Pooled results showed that patients with higher RAR faced a significantly higher risk of mortality (HR 1.88, 95%CI 1.59–2.23). Nonlinearity was observed in the dose-response relationship. Using a reference value of 3 ml/g, each 1 ml/g increase in RAR corresponded to a 27% rise in the mortality HR (HR 1.27, 95%CI 1.16–1.39). Our study demonstrated that elevated RAR values were significantly associated with higher mortality in CVD and exhibited a positive dose-response relationship, suggesting its potential as a novel prognostic biomarker for CVD.

**Supplementary Information:**

The online version contains supplementary material available at 10.1038/s41598-024-81876-z.

## Introduction

Despite advancements in the treatment of cardiovascular disease (CVD), the global cardiovascular burden has risen significantly in the last 20 years, with the mortality rate increasing from 12.1 million in 1990 to 18.6 million in 2019, according to the Global Burden Disease Study 2019. Furthermore, the trend of CVD burden in several countries, which previously had been decreasing, has reversed in the last decade^1^. According to the WHO, CVD was responsible for 32% of all deaths worldwide, with stroke and coronary heart disease accounting for 85% of them^2^. This increase is partly due to an outdated management approach that overlooks individual patient differences by applying uniform treatment protocols^3^. A paradigm shift is needed in CVD management, focusing on incorporating biological parameters, in order to promote more individualized patient care.

Biomarkers, acting as key indicators of physiological and pathological conditions within our bodies, play crucial roles in the cardiovascular field, aiding in early diagnosis, risk stratification, therapeutic assessment, and patient management^4,5^. Effective prognosis stratification is essential for prioritizing the treatment given to patients and determining its intensity. Although current biomarkers have prognostic value, their implementation is limited by cost and accessibility^6^. Many studies have searched for low-cost, widely available, and applicable biomarkers to predict the prognosis of CVD. Among those, red-cell distribution width (RDW) and serum albumin showed promising results. Elevated RDW and decreased serum albumin have been linked to poor outcomes in cardiovascular conditions, offering key prognostic insights^7,8^. Furthermore, recent studies suggest that biomarkers derived from a combination of individual markers yield superior predictive abilities, and researchers have continually hypothesized methods to enhance the accuracy of albumin by incorporating it with other biomarkers, particularly RDW^8,9^. RDW-to-albumin ratio (RAR) has been shown to have a higher prognostication value when compared with commonly used inflammatory markers, such as neutrophil-to-lymphocyte ratio (NLR)^10^and Sequential Organ Failure Analysis (SOFA) score^11^.

Despite several studies linking RAR to mortality in CVD, questions remain regarding the generalizability and robustness of the individual results, and no previous meta-analysis has been done to evaluate this topic comprehensively. Therefore, we undertook a systematic review and meta-analysis to thoroughly assess the role of RAR as a novel prognostic biomarker in CVD, providing a detailed assessment of its prognostic value through dose-response analysis.

## Methods

This meta-analysis followed the standards set by the guidelines of the Preferred Reporting Items for Systematic Reviews and Meta-Analyses (PRISMA) 2020 and was registered in the PROSPERO database (Registration number CRD42023491805)^12^. Ethical clearance was not obtained as this study collected secondary data from published studies.

### Eligibility criteria

The screening process involved evaluating titles and abstracts of gathered studies, adhering to these selection criteria: (1) research including participants aged 18 years or older diagnosed with CVD, including arrhythmia, aortic dissection, cardiomyopathy, congenital heart disease, cor pulmonale, heart failure, ischemic and hemorrhagic stroke, coronary heart disease, peripheral artery disease, pulmonary embolism, pulmonary hypertension, rheumatic heart disease, valvular heart disease, venous thrombosis; (2) RAR value was measured in ml/g; (3) reporting either all-cause or cardiovascular mortality as the primary outcome; (4) the study was conducted under an interventional (randomized or non-randomized trial) or observational (case-control, prospective, and retrospective cohort) study design, and (5) written in English. There were no restrictions on the year of publication. Studies were excluded if they had unavailable full texts, involved non-human subjects, or had overlapping populations/outcomes.

### Search strategy and study selection

IF, CCZ, WW, and PP executed a comprehensive search for studies available up to February 1, 2024, across multiple databases (PubMed, Web of Science, Scopus, ProQuest), complemented by manual and bibliographic searches for further data. Table 1 describes the keyword employed in the search strategy.

**Table 1:** Keywords used in the literature searching.

No	Keywords
1	((Indicator) OR (Predictor) OR (Prognostic) OR (Diagnostic) OR (“Predictive Value”) OR (Mortality) OR (Death) OR (Mortalities) OR (Severity))
2	((“RDW-Albumin Ratio”) OR (“RDW to serum albumin value”) OR (“Red Blood Cell Distribution Width-Albumin ratio”) OR (“Red Cell Distribution Width-to-Albumin ratio”) OR (“Red Cell Distribution Width-Albumin ratio”) OR (“Red Blood Cell Distribution Width-to-Albumin ratio”) OR (“Red Blood Cell Distribution Width”) OR (“Red Cell Distribution Width”))
3	((“Angina pectoris”) OR (Aorta) OR (“Aortic Dissection”) OR (Arrhythmia) OR (Arterial) OR (Arteriosclerosis) OR (Atherosclerosis) OR (Atrial) OR (Atrial Fibrillation) OR (“Cardiac arrest”) OR (Cardiomyopathy) OR (“Cardiovascular Disease”) OR (“Congenital heart disease”) OR (“Cor Pulmonale”) OR (“Coronary Artery Disease”) OR (CVD) OR (“Heart Disease”) OR (“Heart failure”) OR (“Hemorrhagic Stroke”) OR (“Ischemic Heart Disease”) OR (“Ischemic Stroke”) OR (“Myocardial Infarction”) OR (“Peripheral Artery Disease”) OR (“Pulmonary Embolism”) OR (“Pulmonary Hypertension”) OR (“Rheumatic Heart Disease”) OR (Stroke) OR (Thromboembolism) OR (“Valvular heart disease”) OR (Vasculitis) OR (“Venous Thrombosis”) OR (Ventricular))
Final	1 AND 2 AND 3

Following this, duplicates removal and abstract screening were conducted by CCZ and PP. IF, CCZ, WW, and PP independently reviewed the full texts of studies that initially met the criteria. Differences of opinion among the authors were settled through collective discussion.

### Data extraction

Included studies were extracted independently by authors, using a structured table based on the outcome of interest of this study. Recorded data include the following: author’s name, published year, number of participants, demographic characteristics of participants, type of cardiovascular diseases, follow-up period, and patients’ outcome (all-cause mortality (ACM), cardiovascular mortality).

### Quality assessment

The authors independently performed a risk of bias assessment on individual studies. Newcastle-Ottawa Scale (NOS) is utilized to assess the quality of observational studies^13^. Results of the assessment were presented in total scores, which classify studies into poor, fair, and good quality.

### Primary and secondary endpoints

The primary endpoints observed in this study were overall ACM, 30-day ACM, 90-day ACM, 1-year ACM, 3-year ACM, and in-hospital mortality. The secondary endpoints included hospital length of stay and ICU length of stay.

### Statistical analysis

In this study, we used hazard ratios (HR) for mortality outcomes and standardized mean differences (SMD) using Hedges’ g method for length of stay. Mortality outcomes of the highest vs. lowest RAR categories were pooled using the Generic Inverse Variance method, extracting HRs and 95% confidence intervals (CI) from each study. Due to anticipated heterogeneity, a random-effects model was applied. Studies that reported RR as the effect measure were considered equivalent to HR, while studies that reported OR would be converted to HR using a method by Zhang and Yu^14^, as done in previous studies^15,16^. All statistical analyses were carried out in R software (version 4.3.2) using *meta*^17^, *dmetar*^18^, and *dosresmeta*^19^ packages. A two-sided p-value < 0.05 was considered statistically significant.

### High vs. low RAR values

The included studies categorized the RAR values in various ways: above and below the median, per tertiles, and quartiles. To reduce the heterogeneity of the categorization, we assumed the categorization of all included studies to using per tertile and pooled the effects observed from the highest versus the lowest tertile of RAR values, using the method by Danesh et al.^20^. The HRs were log-transformed and then multiplied by the factors of 2.18/2.54 or 2.18/1.59 for studies using quartiles or comparing values above versus below the median, respectively. This method has also been widely used in subsequent studies^21,22^. Heterogeneity in this study was quantified using the Cochran Q (χ^2^) statistic and I^2^ statistic test, where I^2^ of 50% or greater and *p*< 0.10 of the Q statistic represented evidence of significant heterogeneity^23,24^. Given the negligible differences in sample sizes across the included studies, we adopted the Paule-Mandel (PM) method with a Hartung-Knapp (HK) adjustment^25^for estimating between-study variance (tau²), aligning with recent recommendations^26,27^. Subgroup analyses were conducted to investigate the causes of the heterogeneity according to CVD diagnosis and follow-up time (post-hoc).

### Dose-response meta-analysis

Due to the variations in cut-off values for RAR categories across studies, a dose-response meta-analysis was conducted. This employed generalized least-squares regression using the restricted maximum likelihood (REML) method for trend estimation, as suggested by Berlin, Greenland–Longnecker, and Orsini^28,29^. The analysis required the number of cases/person-years, the total number of participants, and the fully adjusted HR with its 95% CI for each category. A one-stage random-effects meta-analysis for aggregated data was employed to include studies with fewer than three RAR categories, with results comparable to the standard two-stage method^30^. Log estimates were then exponentiated to produce the predicted HRs. The dose-response relationship was assessed using a nonlinear, restricted cubic spline (RCS) model using three knots at the 10th, 50th, and 90th percentiles if the Wald test was significant at a two-sided p-value of < 0.10^24^. In cases where the Wald test was not significant or a linear model yielded the lowest Akaike Information Criterion (AIC) value, we reported the relationship using a linear model^31^. Studies that did not report RAR categorization were excluded from this particular analysis.

A non-zero reference group was designated in this analysis. The assigned doses were the mean or median RAR within each reported category. In the absence of these measures, we used the midpoint of the range. For open-ended lower categories, we subtracted the stated lower bound by the adjacent group’s interval width^32^, while for open upper bounds, half of the adjacent interval width was added to the upper bound^15^. For studies reporting only a low versus high group, the doses were set at half the value for the low group and one and a half times the value for the high group, respectively. In cases where complete event data within each category were not available, estimations of missing data were made based on the total case count and the hazard ratio (HR) for every category^33,34^.

### Sensitivity analysis

To ensure the robustness of the results, we undertook sensitivity analyses by performing meta-analyses using alternative methods, such as applying a fixed-effects model and different between-study variance estimators, and excluding the HK adjustment. To identify potential outliers, we conducted a ‘leave-one-out’ sensitivity analysis, recalculating pooled effect sizes and heterogeneity results after sequentially omitting one study at a time^35^. We also repeated the meta-analysis, excluding outliers^24^. Furthermore, to evaluate the influence of different covariates on RAR’s prognostic significance, a meta-regression analysis was conducted employing the REML approach. Publication bias was assessed through funnel plot inspection and Egger’s regression test^36^. If publication bias was detected, the Duval and Tweedie trim and fill approach was employed^37^.

## Results

### Study selection and quality assessment

**Fig. 1:**
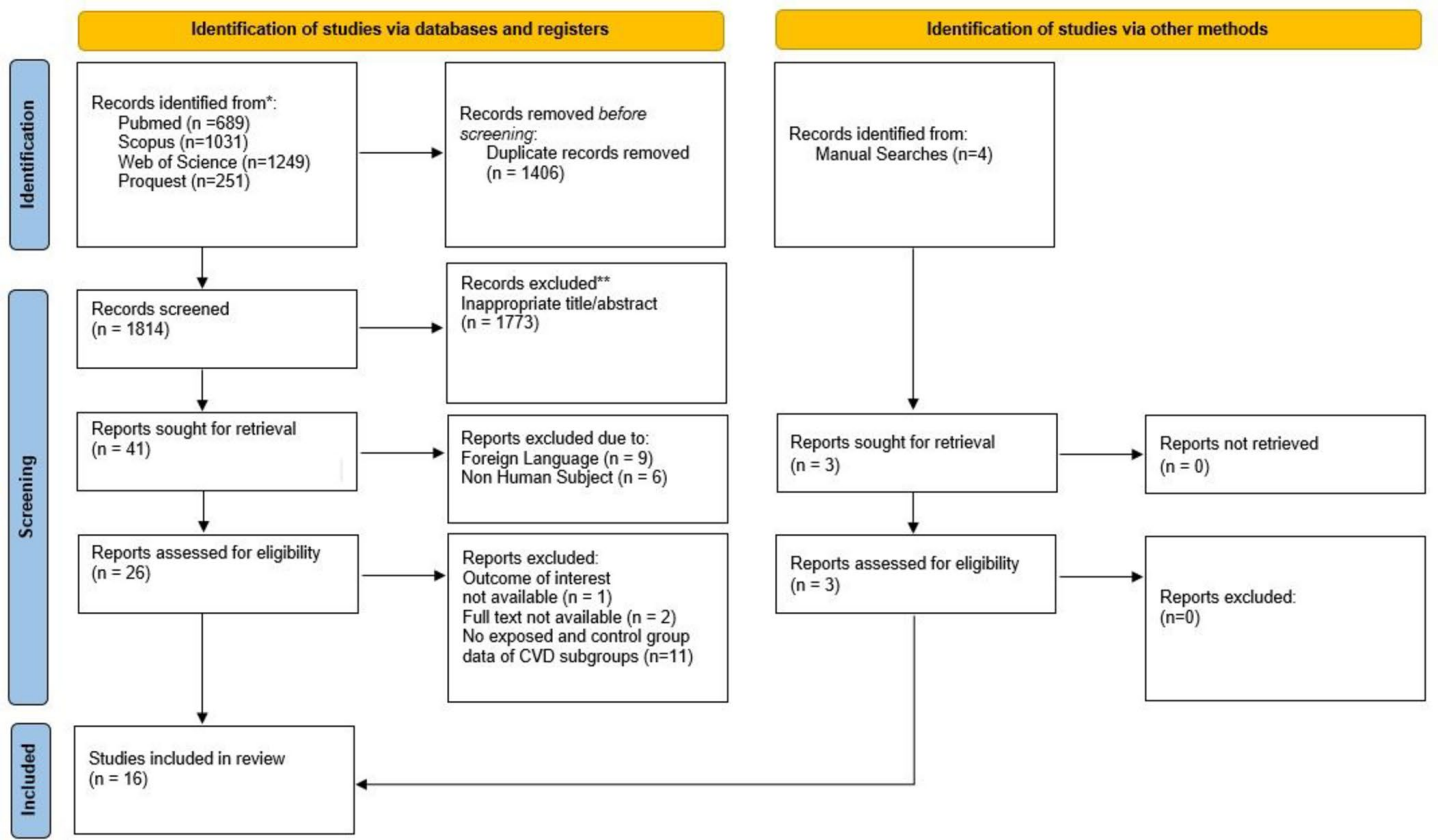
PRISMA flowchart of article selection.

After completing a comprehensive search of databases, 3220 studies were retrieved. Subsequently, 1406 duplicates were removed. Following the screening of titles and abstracts, 15 studies were excluded due to not using English language or involving non-human subjects. Twenty-six studies were screened for eligibility. Studies were excluded for the following reasons: (1) Some included both CVD and non-CVD participants without providing the number of exposed and control groups or effect sizes specifically for the CVD population; (2) In one study, mortality outcome was not reported despite CVD patients being included; and (3) Full texts were unavailable for some studies, preventing eligibility assessment. Three studies were identified through manual searching, resulting in 16 studies included in this meta-analysis (see Fig. 1). All studies were evaluated as being of good quality, with the NOS scale ranging from seven to nine (see Table 2).

### Study characteristics

With a total of 16 studies, 30,933 participants were included in this meta-analysis. All included studies were published between 2021 and 2024 and were classified as cohort studies. Demographically, all studies were conducted in Asia and America: eight studies in the United States and eight in China. Most studies included samples from the ICU with various underlying diseases such as aortic aneurysm, acute myocardial infarction, atrial fibrillation, heart failure, stroke, and pulmonary embolism, or involved patients undergoing certain treatments such as Transcatheter Aortic Valve Replacement (TAVR), Percutaneous Coronary Intervention (PCI), and mechanical thrombectomy. Each study presented different cut-off values and categorizations for RAR. Table 2 outlines the baseline characteristics of the included studies.

### Primary endpoints: mortality outcomes

**Fig. 2:**
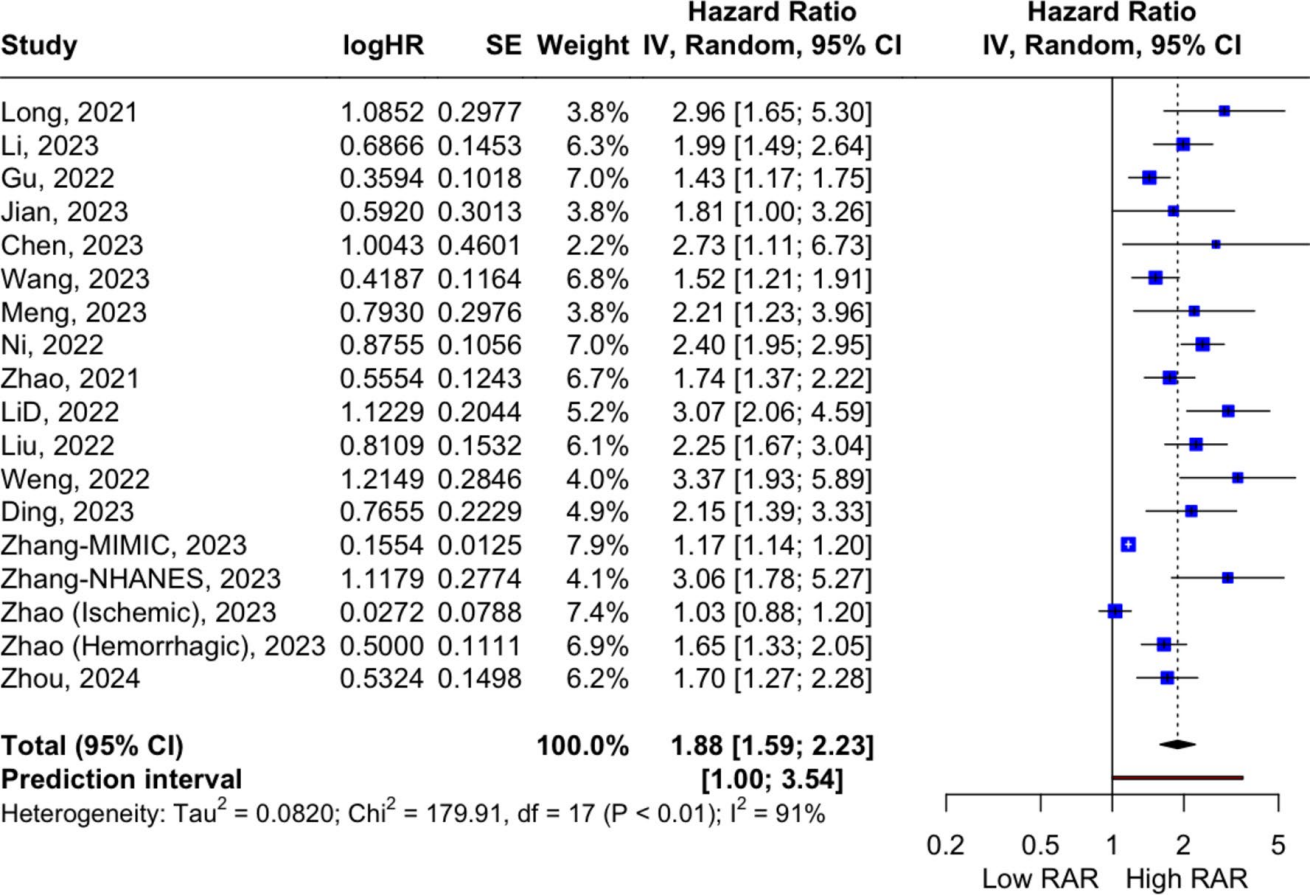
Forest plot of pooled HR for the association between RAR values and ACM (longest follow-up period).

**Fig. 3:**
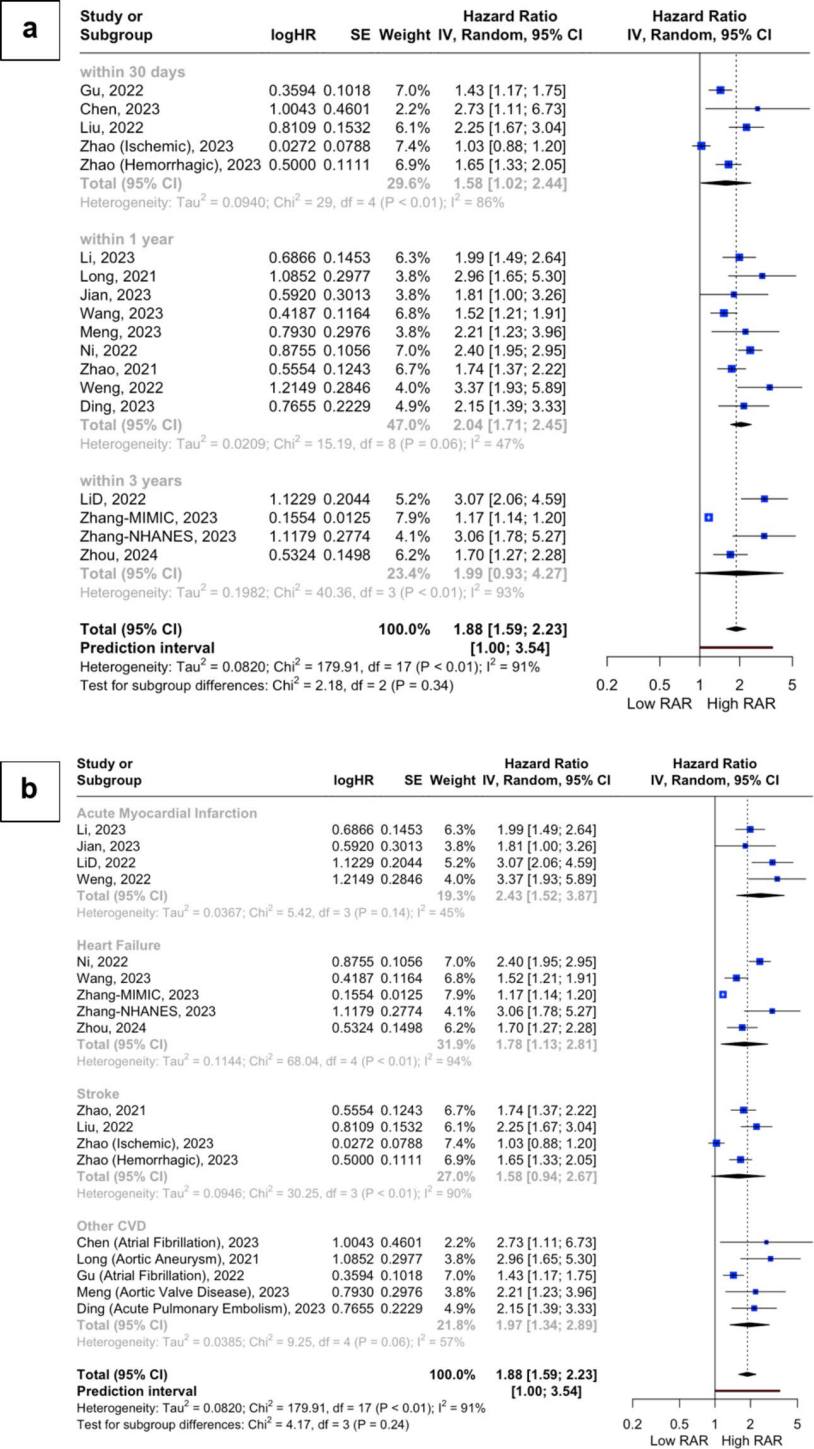
Subgroup analyses of the association between RAR values and overall ACM stratified based on (**a**) follow-up duration and (**b**) CVD diagnoses.

Across all studies, higher RAR values were associated with a greater risk of mortality (HR 1.88, 95%CI 1.59–2.23, *p* < 0.0001, I^2^ = 91%; see Fig. 2). Upon stratifying the analysis for overall ACM by the primary CVD diagnoses, a consistent and significant relationship between RAR values and ACM was observed across all CVD subcategories (*AMI*: HR 2.43, 95%CI 1.52–3.87, I^2^ = 45%; *HF*: HR 1.78, 95%CI 1.13–2.81, I^2^ = 94%; *Stroke*: HR 1.58, 95%CI 0.94–2.67, I^2^ = 90%; *Other CVD*: HR 1.97, 95%CI 1.34–2.89, I^2^ = 57%; p for interaction = 0.24; see Fig. 3). However, in the subgroup analysis stratified by follow-up duration, the association did not reach statistical significance (*Within 30 days*: HR 1.58, 95%CI 1.02–2.44, I^2^ = 86%; *Within one year*: HR 2.04, 95%CI 1.71–2.45, I^2^ = 47%; *Within three years*: HR 1.99, 95%CI 0.93–4.27, I^2^ = 93%; p for interaction = 0.34; see Fig. 3).

**Table 2:** Baseline characteristics of included studies.

Author, Year	Country		Study Design	NOS Scale	Database	Population	Sample size, *n*	Male, *n* (%)	Age, mean ± SD (years)	Outcomes	RAR, mean ± SD (mL/g)	Cut-off values (mL/g)
Long et al., 2021^38^	China		Retrospective cohort	7	MIMIC-III Version 1.4	ICU; Aortic Aneurysm	312	190 (60.3%)	74.9 ± 10.9	30-day ACM, 90-day ACM, 1-year ACM	5.4 ± 1.0	T1: <4.5 T2: 4.5–5.8 T3: >5.8
Li et al., 2023^39^	China		Retrospective cohort	8	MIMIC-III	ICU (> 24 h); AMI over 18 years old	2081	1258 (60.5%)	< 65: 631 (30.3%) ≥ 65: 1450 (69.7%)*	90-day ACM LoS (ICU)	4.32*‡	Low: <4.32 High: ≥4.32
Gu et al., 2022^40^	United States		Retrospective cohort	8	MIMIC-IV Version 1.0	ICU; AF and Sepsis	3042	1782 (58.6%)	74.9 ± 12.3	In-hospital 30-day ACM	5.27 ± 1.5	Q1: <4.06 Q2: 4.06–4.89 Q3: 4.89–6.0 Q4: ≥6.0
Jian et al., 2023^41^†	United States		Retrospective cohort	8	eICU-CRD	ICU; AMI	2594	960 (37%)	66.3 ± 14.1	In-hospital ACM, LoS (ICU), LoS (Hospital)	4.28 ± 1.03	Low: <4.776 High: >4.776
Chen et al., 2023^42^	China		Retrospective cohort	8	Huadong Hospital	Non-ICU; AF 80 years old and over	1,237	528 (46.3%)	84.7 ± 3.9	30-day ACM	3.62 ± 0.37	Low: ≤ 3.37 Middle: 3.37–3.88 High: >3.88
Wang et al., 2023^43^	United States		Retrospective cohort	9	MIMIC-IV Version 2.1	ICU; Acute HF	1432	786 (54.9%)	73.7 ± 14.8	1-year ACM	5.07 ± 1.51	Q1: <4.25, Q2: 4.25–5.29 Q3: >5.29
Meng et al., 2023^44^	China		Retrospective cohort	8	MIMIC-IV Version 2.0	ICU; Patients underwent TAVR	760	402 (52.9%)	82.9 ± 8.2	30-day ACM, 1-year ACM, LoS (ICU), LoS (Hospital)	3.77 ± 0.74	Q1: <3.5 Q2: 3.5–4.0 Q3: >4.0
Ni et al., 2022^45^	China		Retrospective cohort	7	MIMIC-III Version 1.4, Hospital of Wenzhou Medical University	ICU (> 48 h); HF over 18 years old	2841	Q1 1482 (52.18%)	72.7 ± 13.5	30-day ACM, 90-day ACM, 1-year ACM, LoS (ICU), LoS (Hospital)	5.17 ± 1.5	Q1: <4.33 Q2: 4.33–5.44 Q3: >5.44
Zhao et al., 2021^46^	China		Retrospective cohort	7	MIMIC-III Version 1.4	ICU (> 48 h); Stroke over 18 years old (unspecified)	1480	809 (54.7%)	67.7 ± 15.4	30-day ACM, 90-day ACM, 1-year ACM, LoS (ICU)	4.47 ± 1.6	Q1: <3.46 Q2: 3.46–4.03 Q3: 4.03–4.94 Q4: >4.94
Li et al., 2022^47^	China		Retrospective cohort	8	MIMIC-III	ICU; AMI over 16 years old	826	315 (38.1%)	68.3 ± 13.9	30-day ACM, 1-year ACM, 3-year ACM	4.35 ± 1.24	Q1: <3.5 Q2: 3.5–4.0 Q3: 4.0–4.8 Q4: >4.8
Liu et al., 2022^48^†	United States		Retrospective cohort	8	MIMIC-IV, eICU-CRD	ICU; AIS	1412	705 (49.9%)	68.8 ± 15.9	30-day ACM, In-hospital ACM	1.46 ± 0.25	Q1: 0.8–1.2 Q2: 1.2–1.5 Q3: 1.5–2.7
Weng et al., 2022^49^	United States		Retrospective cohort	8	MIMIC-III Version1.4, Hospital of Wenzhou Medical University	ICU; patients post PCI	707	432 (61.1%)	68.2 ± 13.7	30-day ACM, 90-day ACM, 1-year ACM, LoS (ICU)	4.34 ± 1.18	Q1: <3.7 Q2: 3.7–4.5 Q3: >4.5
Zhang et al., 2023^50^†	United States		Retrospective cohort	8	MIMIC-III, NHANES	ICU (MIMIC) and Non-ICU (NHANES); Chronic HF	7758	**NHANES** 965 (51.78%) **MIMIC-III** 3208 (53.32%)	**NHANES** 66.6 ± 5 **MIMIC-III** 73.3 ± 12.4	3-year ACM	**NHANES** 3.47 ± 0.09 **MIMIC-III** 5.36 ± 1.6	**NHANES** Q1: *≤*2.85 Q2: 2.85–3.09 Q3: 3.09–3.1 Q4: >3.1 **MIMIC-III** Q1: <3.78 Q2: 3.78–4.52 Q3: 4.52–5.61 Q4: >5.61
Ding et al., 2023^51^	United States		Retrospective cohort	9	MIMIC-IV	ICU; Acute severe pulmonary embolism	773	395 (51.1%)	61.4 ± 16.9	In-hospital ACM, 1-year ACM	5.7 ± 1.82	Low: 2.68–4.71 Middle: 4.71–6.09 High: 6.09–15.45
Zhao et al., 2023^10^	United States		Retrospective cohort	8	MIMIC-III	ICU (> 24 h); Stroke over 18 years old (hemorrhagic and ischemic)	1601	**Hemorrhagic** 487 (53.63%) **Ischemic** 334 (47.55%)	**Hemorrhagic** 65.7 ± 17.5 **Ischemic** 70.9 ± 21.6	30-day ACM	**Hemorrhagic** 4.12 ± 1.03 **Ischemic** 4.34 ± 1.14	NR
Zhou et al., 2024^52^	China		Retrospective cohort	8	Heart Failure Center of Fuwai Hospital	ICU; Non-Ischemic HF	2077	1380 (66.4%)	52.8 ± 15.7	3-year ACM	3.41 ± 0.7	T1: 3.15 T2: 3.15–3.76 T3: >3.76

AMI: Acute Myocardial Infarction; ICU: Intensive Care Unit; ACM: All-cause mortality; LoS: Length of stay; AF: Atrial Fibrillation; MIMIC: Medical Information Mart for Intensive Care Database; HF: Heart Failure; TAVR: Transcatheter Aortic Valve Replacement; AHF: Acute Heart Failure; AIS: Acute Ischemic Stroke; eICU-CRD: eICU Collaborative Research Database; PCI: Percutaneous Coronary Intervention; NHANES: National Health and Nutrition Examination Survey.

*values are not presented as mean ± SD due to unavailable data.

‡the value is presented as median due to unavailable mean ± SD data.

†effect sizes of the outcomes in the studies were converted into HR.

**Fig. 4:**
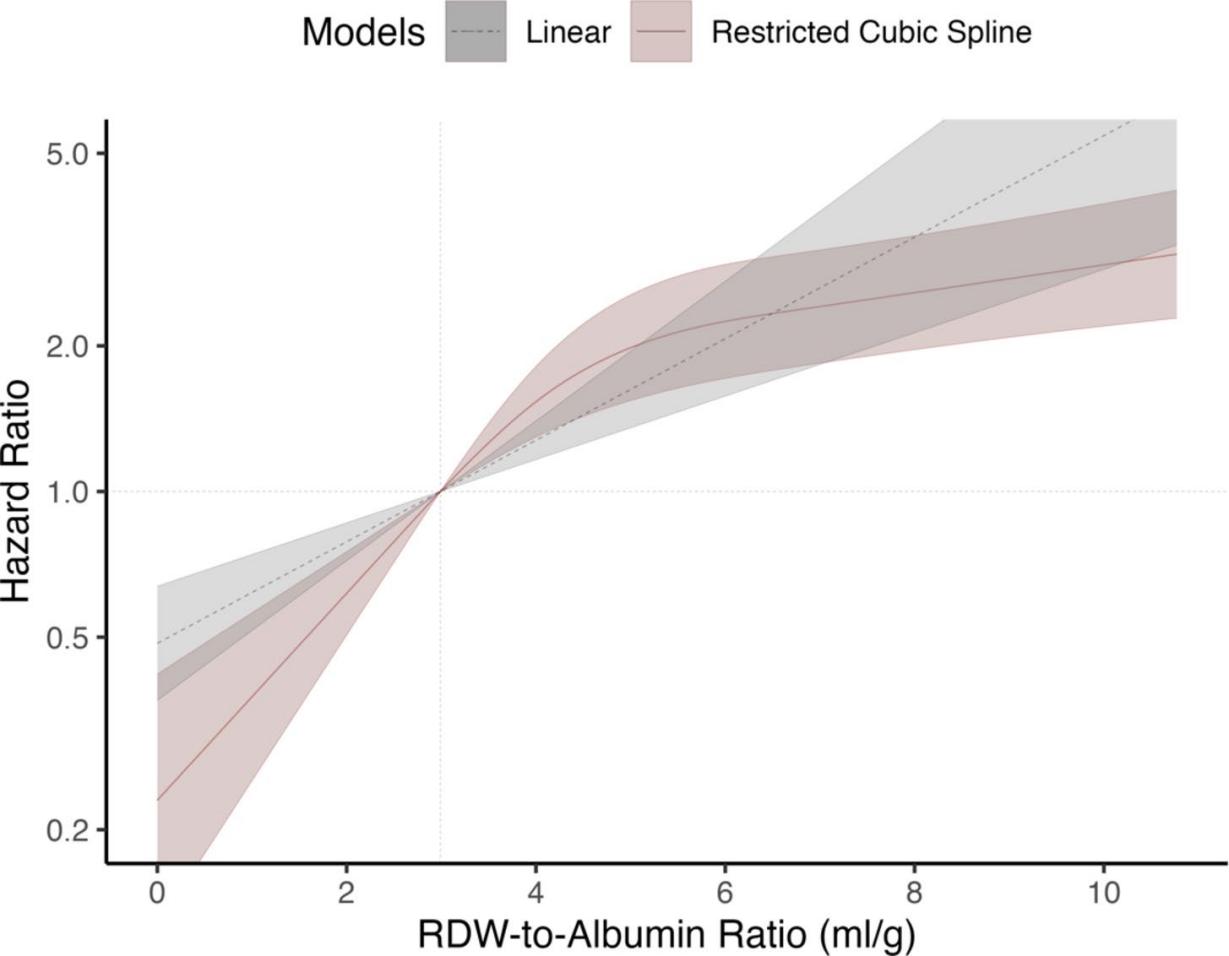
Dose-response curves between RAR values and pooled HR of overall ACM. The relationship is elaborated with two different models: the linear model in grey (dashed line) and the RCS (with knots at 10th, 50th, and 90th percentiles) in red (solid line). The 95%CIs are shaded around each curve.

In the dose-response meta-analysis involving 15 studies with 14,387 participants, estimates were presented per 1 ml/g increment in RAR, with a reference point (HR = 1) set at three ml/g, determined by the weighted mean of all assigned reference doses. Three inputs from two studies lacking RAR categorization were omitted from the analysis^10,50^. We found that the spline model had the lowest AIC value and a significant Wald test result (χ2 = 53, *p* < 0.001), indicating the best fit and nonlinearity. Consequently, the dose-response relationship was primarily illustrated using the RCS model, with the linear model provided for comparison. In the linear model, for each 1 ml/g increase in RAR, the predicted HR for ACM rose by 27% (HR 1.27, 95%CI 1.16–1.39; *p* < 0.0001). The spline model showed a substantial increase in HR from the baseline to approximately 5 ml/g before proceeding to produce a more gradual increase afterward (*RAR 4ml/g*: HR 1.53, 95%CI 1.29–1.80; *RAR 5ml/g*: HR 1.97, 95%CI 1.54–2.53; *RAR 10ml/g*: HR 2.93, 95%CI 2.19–3.91; *p* < 0.0001; see Fig. 4 and Supplementary Table *S3* online).

The pooled random-effects analyses indicated that the in-hospital, 30-day, 90-day, and one-year ACM outcomes were statistically significant, while the 3-year ACM was not (*In-hospital*: HR 1.77, 95%CI 1.20–2.61, I^2^ = 50%, *p* = 0.019; *30-day*: HR 1.92, 95%CI 1.48–2.48, I^2^ = 86%, *p* < 0.001; *90-day*: HR 2.38, 95%CI 1.61–3.53, I^2^ = 59%, *p* = 0.035; *1-year*: HR 2.17, 95%CI 1.73–2.71, I^2^ = 59%, *p* < 0.0001; *3-year*: HR 1.99, 95%CI 0.93–4.27, *p* = 0.064; see Supplementary Table *S2* and *Supplementary Figures **S1*–*5* online).

### Secondary endpoints: length of hospital and ICU stay

The pooled results of the length of hospital (3 studies) and ICU (5 studies) stay were both statistically significant (*Hospital*: SMD 0.62, 95%CI 0.21–1.03, I^2^ = 91%, *p* = 0.022; *ICU*: SMD 0.46, 95%CI 0.93–4.27, *p* = 0.064; see Supplementary Table *S2* and *Supplementary Figures S6*–*S7* online).

### Sensitivity analysis, meta-regression, and publication bias

The leave-one-out analysis of the included studies demonstrated minimal variation in effect sizes and no significant change in its direction and significance, except for two outlying studies (see *Supplementary Figure S8 *online)^10,50^. The pooled results excluding these studies resulted in a slight increase in the effect size and a substantial decrease in the heterogeneity. Subsequently, the meta-analyses were repeated using other methods, and similar effect size, direction, and significance of association were observed (see *Supplementary Table S4* online). The meta-regression analysis revealed no significant association between the modifier variables and ACM (see *Supplementary Table S5*–*S6* online).

Examination of funnel plots visually and analysis through Egger’s test showed a publication bias in the included studies; however, the pooled results after the trim and fill method did not change significantly (HR 1.29, 95%CI 1.00–1.68; *p* < 0.05; I^2^ = 91%; see Supplementary Table *S2* and *Supplementary Figure S9*–*S13* online).

## Discussion

This meta-analysis is the first to explore the relationship between RAR and mortality in CVD patients, revealing a significant correlation between higher RAR and increased mortality, as well as longer hospital and ICU stays. A nonlinear dose-response relationship was found, with results adjusted for confounding factors. Subgroup and sensitivity analyses confirmed the robustness of the findings despite observed heterogeneity.

Previous studies on RDW and albumin levels in CVD populations showed significant but varied predictive values. RDW exhibited a linear relationship with mortality (HR 1.03 per unit increase)^34^, while albumin showed a J-shaped association^53^. Our analysis found that RAR provided a stronger and more consistent predictive value (HR 1.27 per ml/g increase) than RDW or albumin alone.

RDW, which measures red blood cell size variation, is a recognized biomarker for inflammation. Elevated RDW levels indicate impaired erythropoiesis, commonly due to chronic inflammation, anemia, or nutritional deficiency, all frequent conditions in CVD^54^. These factors disrupt bone marrow function, decrease erythropoietin synthesis, and lead to anisocytosis and adverse cardiac remodeling^7,55^. Serum albumin serves as a multifunctional circulatory protein with antioxidant, anti-inflammatory, anticoagulant, and anti-platelet aggregation activities that are crucial in the pathophysiology of CVD. Hypoalbuminemia reflects conditions like malnutrition, inflammatory syndromes, and hypervolemia. However, unlike RDW, hypoalbuminemia can also worsen CVD conditions by increasing oxidative stress, endothelial dysfunction, and thrombogenic risks^9,56^. Thus, the combination of these markers may enhance CVD prognostication through better detection of underlying inflammation, oxidative stress, and increased atherothrombotic risk.

Distinct differences in effect sizes exist among specific cardiovascular diseases. AMI exhibited the highest hazard ratio, with a 2.4-fold increased likelihood of mortality (HR 2.43, 95%CI 1.52–3.87, I^2^= 45%). Beyond the previously stated mechanisms, this could be attributed to alterations in microvascular flow due to elevated RDW^55^, as well as increased thrombus formation and myocardial edema from hypoalbuminemia in a hyperacute setting^9,56^. In comparison, heart failure and stroke had lower HRs but still indicated increased risks. Oxidative stress in stroke may impair erythropoiesis, reducing cerebral tissue reperfusion^57^, while hypoalbuminemia may blunt the anti-inflammatory response^58^. In heart failure, iron deficiency and neurohormonal activation may cause anisocytosis, increasing cardiovascular stress^59^. Hypoalbuminemia may also raise the risk of pulmonary edema and diuretic resistance^60^. The variability in effects across CVDs (Chi² = 4.2, p for interaction = 0.2) highlights the multifaceted nature of CVD pathogenesis. An elevated RAR could prompt more intensive monitoring and earlier, aggressive interventions to prevent mortality, such as ICU admission and treatment intensification.

RAR demonstrated the strongest predictive value for mortality among hematologic biomarkers in CVD populations (HR 1.88), outperforming WBC count (HR 1.64), NLR (HR 1.14 per quartile increase), and hemoglobin levels (HR 0.92)^61–63^. These findings underscore RAR’s robustness as a mortality predictor among commonly used markers in CVD populations.

Despite numerous studies exploring hematological parameters in CVDs^56,59^, varying cut-off values often introduce interpretation bias. Dose-response meta-analysis offers a novel approach by interpreting RAR as a continuous variable (per 1 ml/g increase) rather than relying solely on categorical groupings, thus mitigating this bias^64^. Our study demonstrated a significant dose-response relationship between RAR and mortality in CVD populations. Since RAR is derived from routine hematological tests, establishing precise cut-off values for each specific CVD is crucial for its practical application in clinical settings.

This study has several limitations. First, heterogeneity in the analysis could introduce bias, necessitating subgroup and sensitivity analyses. The variability in RAR cut-off values and categorizations among studies may have influenced the findings, though we addressed this through a dose-response meta-analysis. Additionally, several studies were excluded due to unavailable CVD-specific data, potentially causing a publication bias. Lastly, as all studies were conducted in Asia and America, the generalizability of the findings to other regions may be limited.

Several potential sources of heterogeneity in our study were identified. First, variations in CVD types and differences in RAR values across studies likely contributed to the heterogeneity, which we addressed through subgroup analysis based on CVD diagnoses and dose-response analysis. Second, since all studies were cohort designs with expected methodological variability and potential confounders, fully adjusted effect sizes were used. Third, after conducting a leave-one-out meta-analysis and using the *find.outliers* function in Rstudio, outliers were identified within the included studies. Excluding these outliers in sensitivity analyses reduced heterogeneity substantially from 91 to 58%. Finally, although Egger’s regression test indicated publication bias and small-study effects, trim-and-fill analysis confirmed the results remained significant.

As the field evolves, it is imperative to recognize the need for future research and potential guideline development based on standardized approaches. The emphasis on prospective studies to determine causality, along with further research involving larger, well-defined, or homogeneous cohorts, is essential to solidify the role of RAR as a reliable biomarker for predicting mortality in CVD patients. Additionally, meta-analyses of the association between RAR and mortality outcomes across different CVDs are crucial to further establish its clinical relevance.

## Conclusion

This systematic review and dose-response meta-analysis represent the initial study to illustrate a noteworthy association between elevated RAR values and increased mortality risk and length of stay outcomes, along with a significant positive dose-response relationship among CVD populations. These findings advocate for incorporating RAR into routine clinical evaluations, highlighting its potential role as a cost-effective and accessible prognostic tool for mortality risk assessment in patients with CVD.

Future research is needed to confirm these results and further investigate the potential link between RAR and all-cause mortality in CVD.

## Electronic supplementary material

Below is the link to the electronic supplementary material.

Supplementary Material 1

## Data Availability

The datasets generated and analyzed during this study are not available for public access due to restrictions associated with intellectual property rights. However, these datasets are accessible upon request to the corresponding authors. Interested parties are encouraged to reach out to the corresponding authors directly to discuss the terms under which access to the datasets may be granted.
