# Supplementary material for: A systematic review and dose-response meta-analysis of red blood cell distribution width to albumin ratio as mortality predictor in cardiovascular disease
Source: Sci Rep. 2025 Sep 1;15:32053. doi: 10.1038/s41598-024-81876-z (PMC12402460; doi:10.1038/s41598-024-81876-z)
Supplement: Supplementary file 1 — Supplementary Material 1 [file 41598_2024_81876_MOESM1_ESM.pdf]

Red Blood Cell Distribution Width-to-Albumin Ratio as Novel Biomarker Predicting Mortality in Cardiovascular Disease: A Systematic Review and Dose-Response Meta Analysis

Supplementary Materials

Table of Contents

Primary Endpoints 1

Table S1. Summary of pooled outcomes 1

Table S2. Dose-response associations between RAR values and mortality 1

Figure S1. Forest plot of 30-day mortality pooled HR using random-effect model (Zhao 2021 removed due to overlapping population and outcome with Zhao 2023) 2

Figure S2. Forest plot of 90-day mortality pooled HR using random-effect model 2

Figure S3. Forest plot of 1-year mortality pooled HR using random-effect model 3

Figure S4. Forest plot of 3-year mortality pooled HR using random-effect model 3

Figure S5. Forest plot of in-hospital mortality pooled HR using random-effect model ... 3

Secondary Endpoints 4

Figure S6. Forest plot of length of ICU stay using random-effect model 4

Figure S7. Forest plot of length of hospital stay using random-effect model 4

Sensitivity Analysis 5

Table S3. Results comparison of different meta-analysis models and between-study variance estimators 5

Figure S8. Leave-one-out meta-analysis 6

Meta-regression 7

Table S4. Data used for meta-regression analysis* 7

Table S5. Summary of meta-regression analysis 7

Publication Bias 8

Figure S9. Funnel plot of overall mortality pooled HR using random-effect model 8

Figure S10. Funnel plot of 30-day mortality pooled HR using random-effect model 8

Figure S11. Funnel plot of 90-day mortality pooled HR using random-effect model 9

Figure S12. Funnel plot of 1-year mortality pooled HR using random-effect model 9

Figure S13. Funnel plot of 3-year mortality pooled HR using random-effect model 10

PRISMA 2020 Checklist 11

Table S6. PRISMA 2020 Checklist 11

PRIMARY ENDPOINTS

Table S1. Summary of pooled outcomes

No	Outcomes	No. of inputs/studies	Effect measures	EM (95%CI)	p value	Heterogeneity			Publication Bias
						I^2	tau^2	p value	
1	Overall ACM	18	HR	1.88 (1.59 - 2.23)	<0.0001	91%	0.082	<0.001	0.001
2	Adjusted Overall ACM*	16		2.02 (1.75 - 2.32)	<0.0001	59%	0.034	<0.001	0.01
3	30-day ACM	10		1.92 (1.48 - 2.48)	<0.001	86%	0.062	<0.001	
4	90-day ACM	5		2.38 (1.61 - 3.53)	0.035	59%	0.056	<0.001	
5	1-year ACM	8		2.17 (1.73 - 2.71)	<0.0001	59%	0.035	<0.001	
6	3-year ACM	4		1.99 (0.93 - 4.27)	0.064	93%	0.198	<0.001	
7	In-hospital ACM	4		1.77 (1.20 - 2.61)	0.019	50%	0.025	<0.001	
8	Length of hospital stay	3	SMD	0.62 (0.21 - 1.03)	0.022	91%	0.015	<0.001	
9	Length of ICU stay	5		0.46 (0.14 - 0.77)	0.015	95%	0.047	<0.001	

Abbreviations: ACM, All-cause mortality; SMD, Standardized Mean Difference; EM, Effect measures
*Removed Zhang-MIMIC, 2023 and Zhao (Ischemic), 2023

Table S2. Dose-response associations between RAR values and mortality

**RAR (ml/g)**	**Predicted Hazard Ratio (95%CI)**	
	**Linear**	**Spline**
**0**	0.48 (0.37 - 0.64)	0.23 (0.13 - 0.42)
**1**	0.62 (0.51 - 0.74)	0.37 (0.25 - 0.56)
**2**	0.79 (0.72 - 0.86)	0.61 (0.50 - 0.75)
**3**	**Reference**	
**4**	1.27 (1.16 - 1.39)	1.53 (1.29 - 1.80)

**5**	1.62 (1.35 - 1.94)	1.97 (1.54 - 2.53)
**6**	2.06 (1.57 - 2.71)	2.24 (1.71 - 2.93)
**7**	2.63 (1.83 - 3.78)	2.40 (1.84 - 3.13)
**8**	3.35 (2.13 - 5.27)	2.56 (1.96 - 3.36)
**9**	4.26 (2.47 - 7.34)	2.74 (2.08 - 3.61)
**10**	5.43 (2.88 - 10.24)	2.93 (2.19 - 3.91)

Figure S1. Forest plot of 30-day mortality pooled HR using random-effect model (Zhao 2021 removed due to overlapping population and outcome with Zhao 2023)

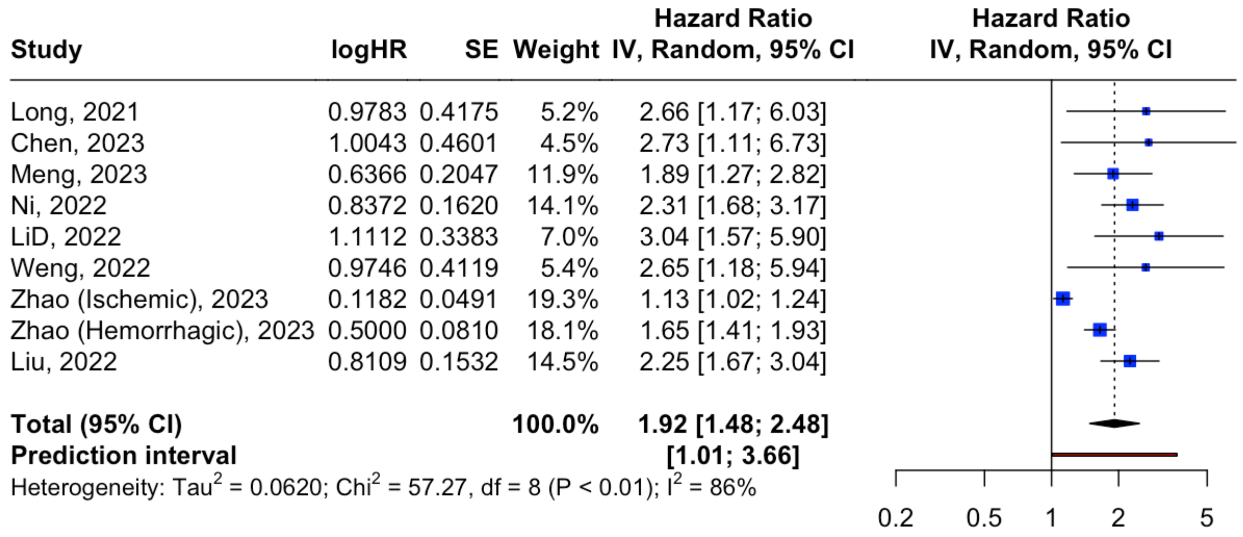

Figure S2. Forest plot of 90-day mortality pooled HR using random-effect model

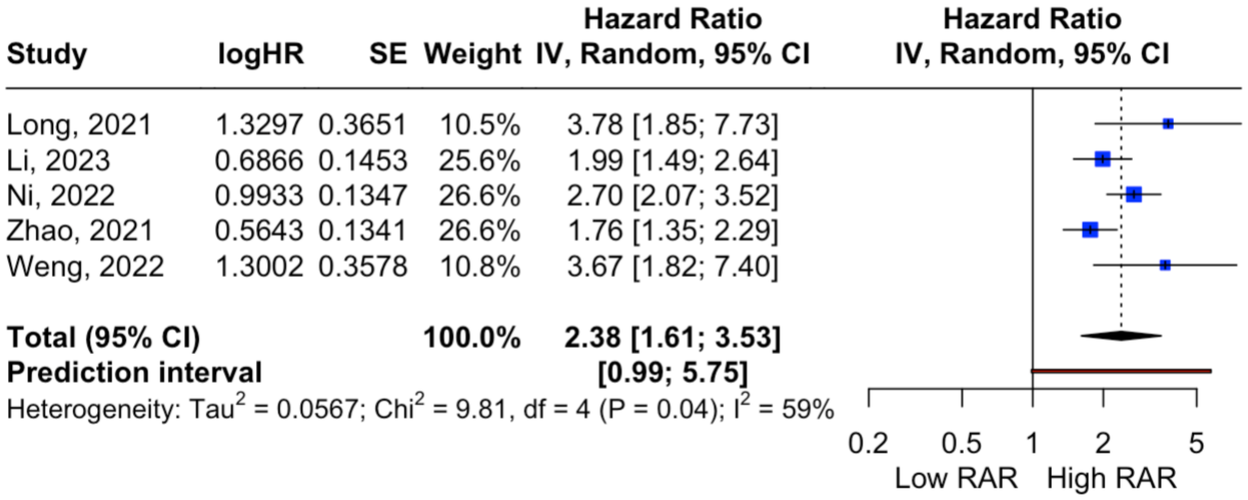

Figure S3. Forest plot of 1-year mortality pooled HR using random-effect model

Study	logHR	SE	Weight	Hazard Ratio IV, Random, 95% CI
Long, 2021	1.0852	0.2977	7.3%	2.96 [1.65; 5.30]
Wang, 2023	0.4187	0.1164	18.6%	1.52 [1.21; 1.91]
Meng, 2023	0.7930	0.2976	7.3%	2.21 [1.23; 3.96]
Ni, 2022	0.8755	0.1056	19.6%	2.40 [1.95; 2.95]
Zhao, 2021	0.5554	0.1243	17.9%	1.74 [1.37; 2.22]
LiD, 2022	1.0299	0.2227	10.7%	2.80 [1.81; 4.33]
Weng, 2022	1.2149	0.2846	7.8%	3.37 [1.93; 5.89]
Ding, 2023	0.7655	0.2229	10.7%	2.15 [1.39; 3.33]
**Total (95% CI)**			**100.0%**	**2.17 [1.73; 2.71]**
**Prediction interval**				**[1.30; 3.62]**

Heterogeneity: $Tau^2 = 0.0351$; $Chi^2 = 17.18$, df = 7 (P = 0.02); $I^2 = 59\%$

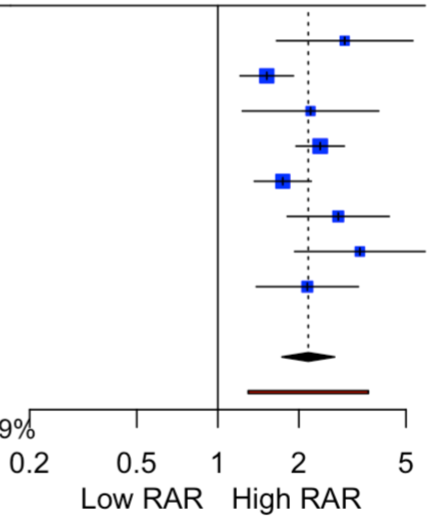

Figure S4. Forest plot of 3-year mortality pooled HR using random-effect model

Study	logHR	SE	Weight	Hazard Ratio IV, Random, 95% CI
LiD, 2022	1.1229	0.2044	24.0%	3.07 [2.06; 4.59]
Zhang-MIMIC, 2023	0.1554	0.0125	29.0%	1.17 [1.14; 1.20]
Zhang-NHANES, 2023	1.1179	0.2774	20.9%	3.06 [1.78; 5.27]
Zhou, 2024	0.5324	0.1498	26.1%	1.70 [1.27; 2.28]
**Total (95% CI)**			**100.0%**	**1.99 [0.93; 4.27]**

Heterogeneity: $Tau^2 = 0.1982$; $Chi^2 = 40.36$, df = 3 ($P < 0.01$); $I^2 = 93\%$

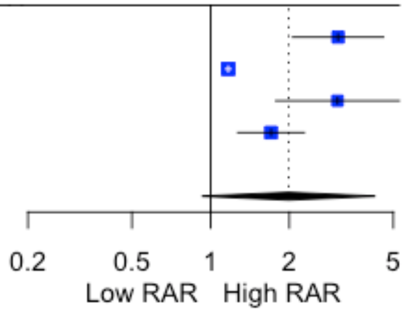

Figure S5. Forest plot of in-hospital mortality pooled HR using random-effect model

Study	logHR	SE	Weight	Hazard Ratio IV, Random, 95% CI
Gu, 2022	0.3594	0.1018	41.5%	1.43 [1.17; 1.75]
Jian, 2023	0.5920	0.3013	12.9%	1.81 [1.00; 3.26]
Liu, 2022	0.6259	0.1690	27.6%	1.87 [1.34; 2.60]
Ding, 2023	0.9478	0.2397	18.0%	2.58 [1.61; 4.13]
**Total (95% CI)**			**100.0%**	**1.77 [1.20; 2.61]**
**Prediction interval**				**[0.74; 4.22]**

Heterogeneity: $Tau^2 = 0.0259$; $Chi^2 = 6.05$, df = 3 (P = 0.11); $I^2 = 50\%$

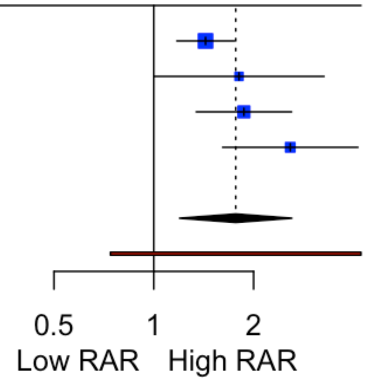

SECONDARY ENDPOINTS

Figure S6. Forest plot of length of ICU stay using random-effect model

Study	Experimental Mean	Experimental SD	Experimental Total	Control Mean	Control SD	Control Total	Weight	Std. Mean Difference IV, Random, 95% CI
Li, 2022	8.55	8.3500	543	5.97	6.9100	1538	20.8%	0.35 [0.25; 0.45]
Jian, 2023	6.39	6.5411	318	3.32	2.9238	2276	20.3%	0.86 [0.74; 0.98]
Ni, 2022	5.95	7.1300	950	4.55	5.3000	938	20.9%	0.22 [0.13; 0.31]
Zhao, 2021	8.00	9.1000	370	5.30	7.0000	369	19.6%	0.33 [0.19; 0.48]
Weng, 2022	6.80	8.7000	236	3.20	4.2000	233	18.4%	0.53 [0.34; 0.71]
**Total (95% CI)**			**2417**			**5354**	**100.0%**	**0.46 [0.14; 0.77]**
**Prediction interval**								**[-0.31; 1.22]**

Heterogeneity: $Tau^2 = 0.0472$; $Chi^2 = 74.78$, df = 4 ($P < 0.01$); $I^2 = 95\%$

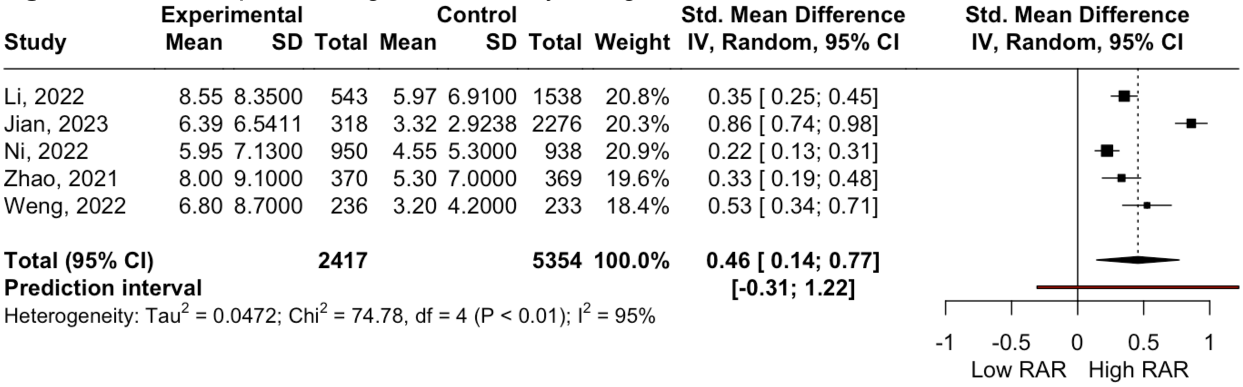

Figure S7. Forest plot of length of hospital stay using random-effect model

Study	Experimental Mean	Experimental SD	Experimental Total	Control Mean	Control SD	Control Total	Weight	Std. Mean Difference IV, Random, 95% CI
Jian, 2023	12.53	10.4201	812	6.90	5.6762	1782	36.8%	0.75 [0.67; 0.84]
Meng, 2023	7.70	5.9600	253	4.35	3.7200	254	26.9%	0.67 [0.49; 0.85]
Ni, 2022	14.14	12.7000	950	9.50	7.2400	938	36.3%	0.45 [0.36; 0.54]
**Total (95% CI)**			**2015**			**2974**	**100.0%**	**0.62 [0.21; 1.03]**
**Prediction interval**								**[-1.27; 2.51]**

Heterogeneity: $Tau^2 = 0.0157$; $Chi^2 = 23.13$, df = 2 ($P < 0.01$); $I^2 = 91\%$

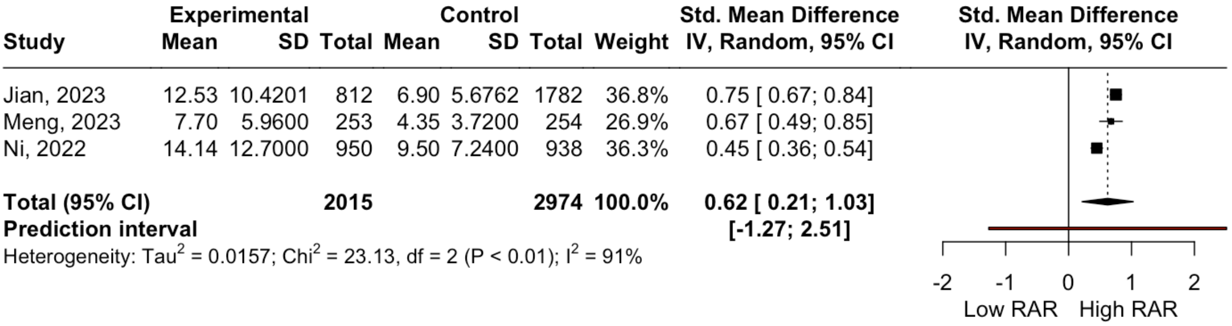

SENSITIVITY ANALYSIS

Table S3. Results comparison of different meta-analysis models and between-study variance estimators

Models	Between-study variance estimators	Hazard Ratio (18 inputs from 16 studies)	Heterogeneity			Adjusted* Hazard Ratio (16 inputs from 16 studies)	Heterogeneity		
		TE (95%CI)	I^2	tau^2	p	TE (95%CI)	I^2	tau^2	p
Random Effects - IV	PM + HK adjustment (default)	1.88 (1.59 - 2.23)	91 %	0.082	<0.0001	2.02 (1.75 - 2.32)	58.6 %	0.0340	<0.0001
	DL + HK adjustment	1.90 (1.61 - 2.26)		0.107	<0.0001	2.02 (1.75 - 2.32)		0.036	<0.0001
	REML + HK adjustment	1.89 (1.59 - 2.24)		0.082	<0.0001	2.02 (1.76 - 2.33)		0.037	<0.0001
Fixed Effects - IV	PM + HK adjustment	1.22 (1.19 - 1.25)		0.0820	<0.0001	1.89 (1.75 – 2.05)		0.0340	<0.0001

*Removed Zhang-MIMIC, 2023 and Zhao (Ischemic), 2023

Figure S8. Leave-one-out meta-analysis

Study	Hazard Ratio IV, Random, 95% CI
Omitting Long, 2021	1.85 [1.55; 2.20]
Omitting Li, 2023	1.88 [1.57; 2.26]
Omitting Gu, 2022	1.92 [1.61; 2.30]
Omitting Jian, 2023	1.89 [1.58; 2.26]
Omitting Chen, 2023	1.87 [1.57; 2.23]
Omitting Wang, 2023	1.92 [1.60; 2.29]
Omitting Meng, 2023	1.88 [1.57; 2.24]
Omitting Ni, 2022	1.85 [1.55; 2.21]
Omitting Zhao, 2021	1.90 [1.58; 2.28]
Omitting LiD, 2022	1.82 [1.54; 2.16]
Omitting Liu, 2022	1.87 [1.56; 2.23]
Omitting Weng, 2022	1.83 [1.55; 2.16]
Omitting Ding, 2023	1.88 [1.57; 2.24]
Omitting Zhang-MIMIC, 2023	1.95 [1.65; 2.30]
Omitting Zhang-NHANES, 2023	1.84 [1.55; 2.18]
Omitting Zhao (Ischemic), 2023	1.95 [1.67; 2.28]
Omitting Zhao (Hemorrhagic), 2023	1.91 [1.59; 2.29]
Omitting Zhou, 2024	1.90 [1.59; 2.28]
**Total (95% CI)**	**1.88 [1.59; 2.23]**

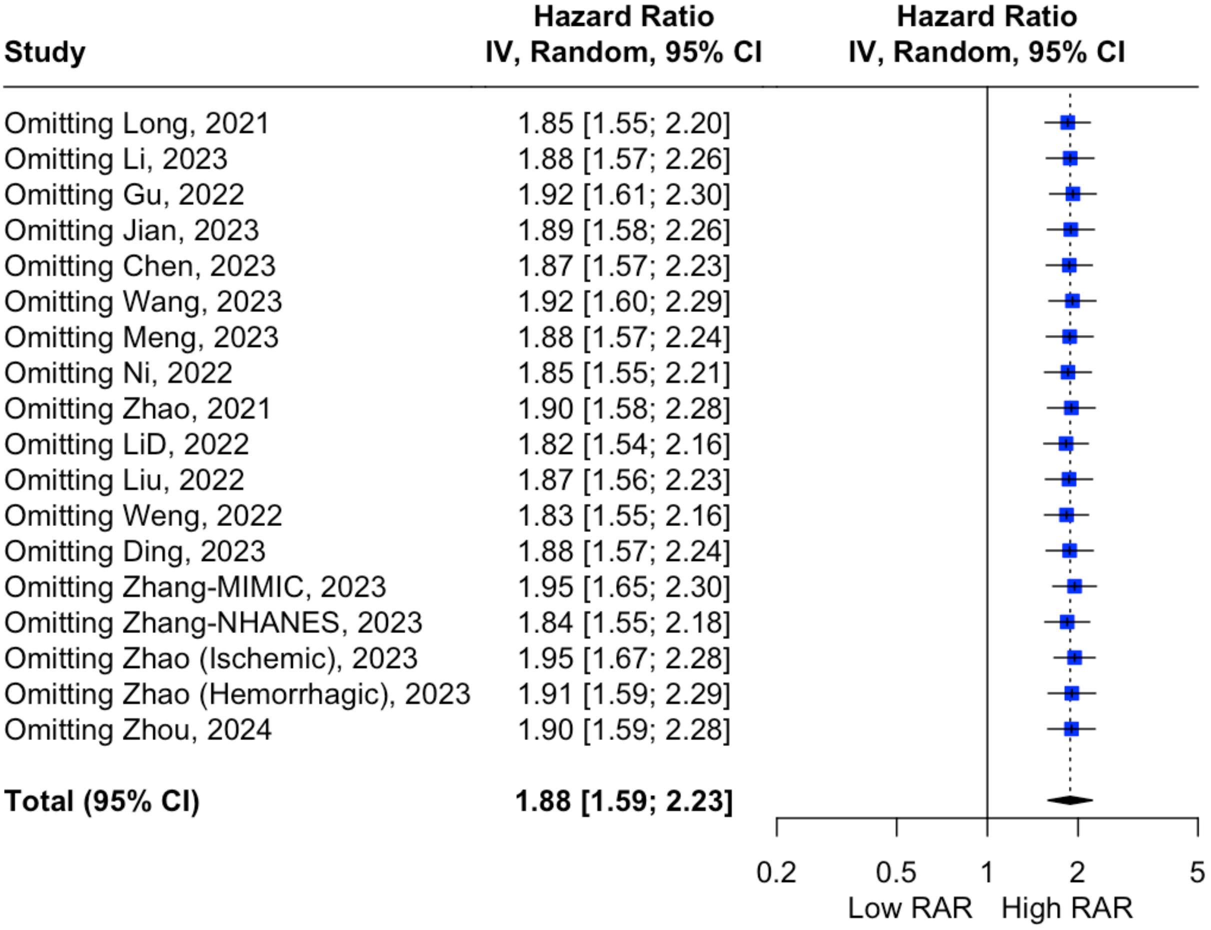

META-REGRESSION

The missing data are imputed using the classification and regression trees (CART) method (Burgette and Reiter *et al.*, 2010). The imputation is performed in R ver. 4.3.2 using MICE package. The following are results from the imputation:

Table S4. Data used for meta-regression analysis*

Author	Follow-up	Population	Age	Male	HR	RR	SBP	Hb	WBC	HCT	BUN	SCr
Long, 2021	within 3 years	208	73.95	61.53	82.53	18.91	117.52	10.90	12.95	32.55	29.07	1.57
Li, 2023	within 3 years	2081	67.3	60.5	88.3	19	122	11.9	12.6	36.09	33	1.8
Gu, 2022	within 30 days	1522	74.2	62.5	93.2	19.05	121	11.2	12.60	34.5	29	1.35
Jian, 2023	within 3 years	2594	66.35	37	86.43	19	112.38	13.1	11.2	33.49	23.5	1.26
Chen, 2023	within 30 days	753	84.7	46.3	85.23	21.7	133.7	11.91	7.25	34.5	21	1.04
Wang, 2023	within 3 years	953	73.35	55.83	92.45	21.12	120.75	10.92	11.79	33.96	28.5	1.25
Meng, 2023	within 3 years	507	82.95	52.9	92.45	21.7	121	11	7	33.9	24	1.1
Ni, 2022	within 3 years	1888	72	55.44	84.84	19.94	114.75	9.85	10.57	29.67	36.43	1.88
Zhao, 2021	within 3 years	739	66.3	55.07	83.61	19.05	125.09	12.4	14.7	36.84	27.61	1.5
LiD, 2022	within 3 years	411	66.89	36.74	85.23	18.71	111.27	11.89	12.56	33.9	28	1.4
Liu, 2022	within 30 days	943	67.3	49.2	86.43	19.8	117.52	12.03	11.63	36.69	25.78	1.45
Weng, 2022	within 3 years	469	53.82	63.11	80.52	18.45	112.38	10.94	10.85	31.92	24.54	1.35
Ding, 2023	within 3 years	515	61.43	51.1	97.8	21.7	124.94	10.88	11.96	29.67	21	0.95
Zhang-MIMIC, 2023	within 3 years	6016	74.28	53.3	93.2	19.05	111.27	11.03	12.16	33.49	40.69	2.06
Zhang-NHANES, 2023	within 3 years	1742	66.65	51.78	83.61	21.12	125.09	13.7	7.67	40.66	20.04	1.35
Zhao (Ischemic), 2023	within 30 days	693	70.9	48.19	93.2	18.31	135.79	12.15	12.95	36.09	25	1.2
Zhao (Hemorrhagic), 2023	within 30 days	908	65.68	53.3	92.45	17.87	139.92	12.98	14.7	38.02	19.5	0.975
Zhou, 2024	within 3 years	2077	52.77	66.4	92.45	17.87	139.92	14.01	14.7	42.05	33	1.8

*Values colored red were the imputed missing data

Table S5. Summary of meta-regression analysis

Variables	Effect estimates (18 inputs from 16 studies)	
	REML + Hartung-Knapp	REML w/o Hartung-Knapp
Population	0.0750	0.0720
Age	0.4336	0.4310
Follow-up	0.1493	0.1453
HCT	0.4750	0.4804
Hemoglobin	0.7585	0.7628
BUN	0.2751	0.2816
SCr	0.7432	0.7485

Abbreviation: HR, heart rate; RR, respiratory rate; SBP, systolic blood pressure; Hb, haemoglobin; WBC, white blood cells; HCT, haematocrit; BUN, Blood Urea Nitrogen; SCr, Serum creatinine

PUBLICATION BIAS

Figure S9. Funnel plot of overall mortality pooled HR using random-effect model

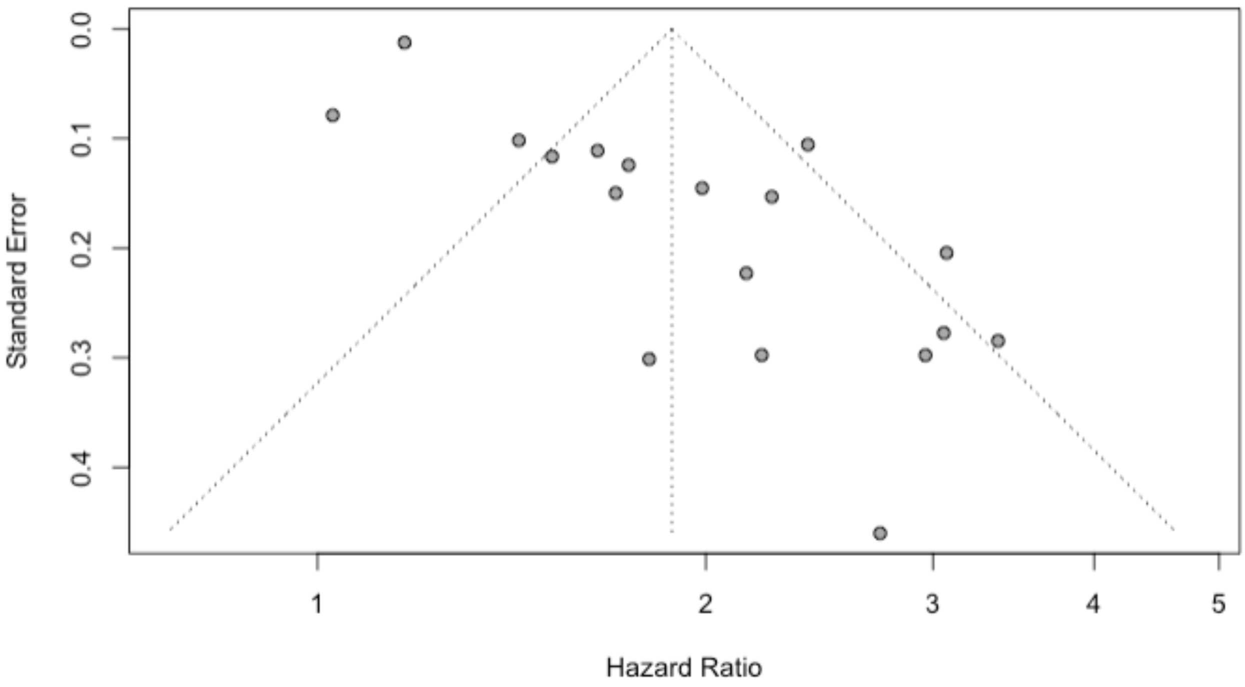

Figure S10. Funnel plot of 30-day mortality pooled HR using random-effect model

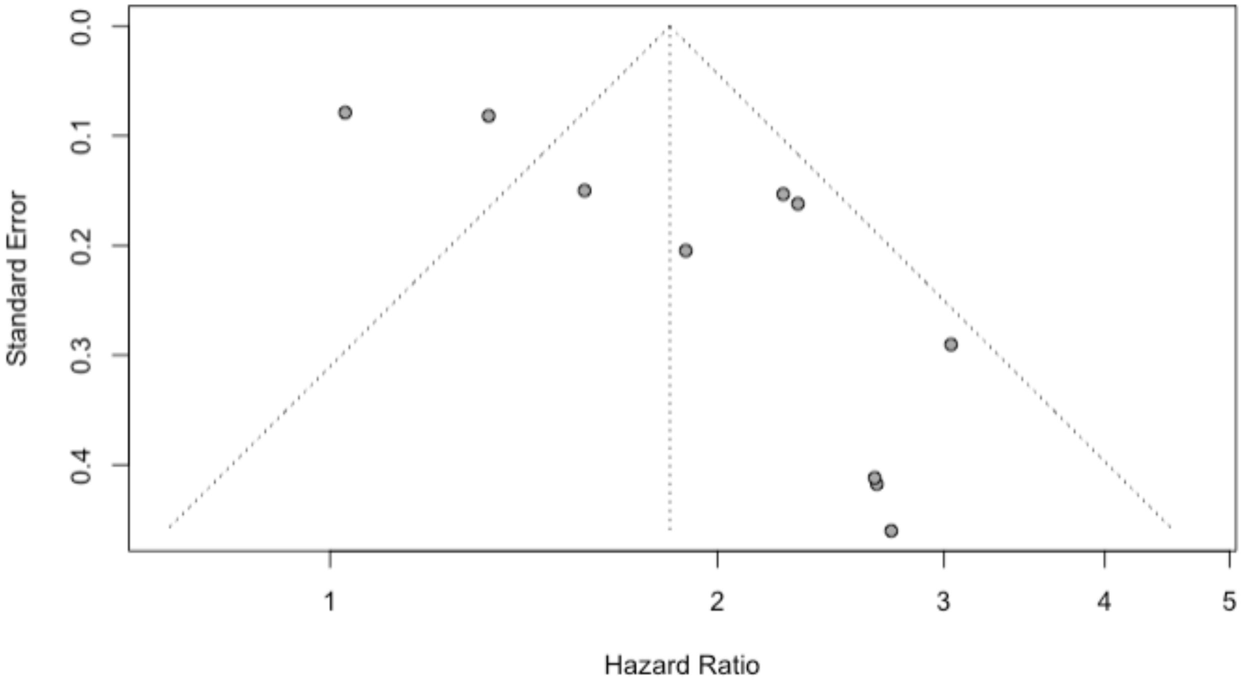

Figure S11. Funnel plot of 90-day mortality pooled HR using random-effect model

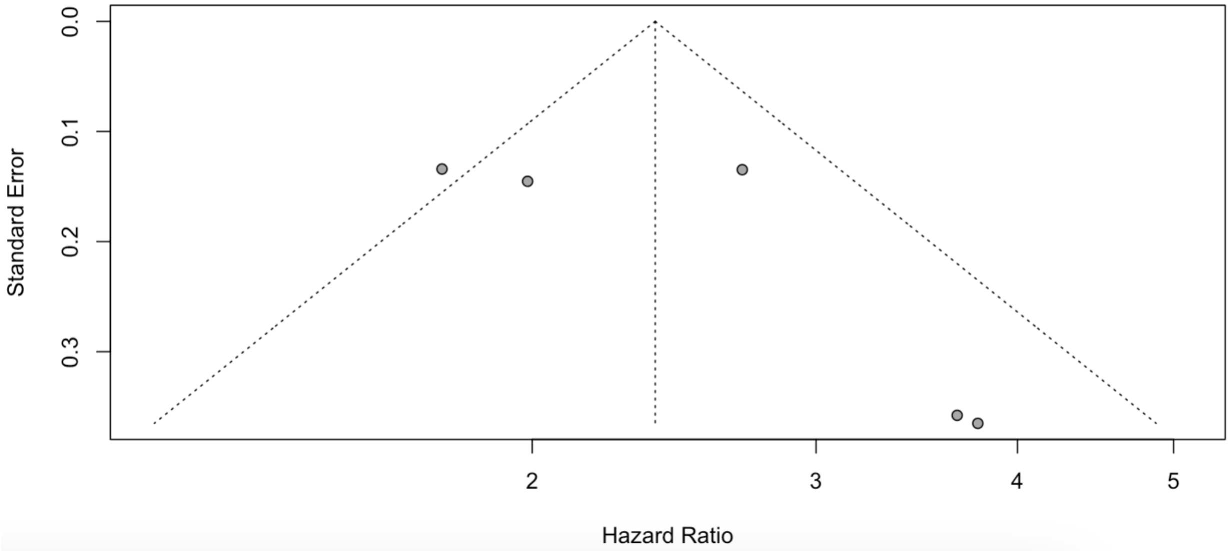

Figure S12. Funnel plot of 1-year mortality pooled HR using random-effect model

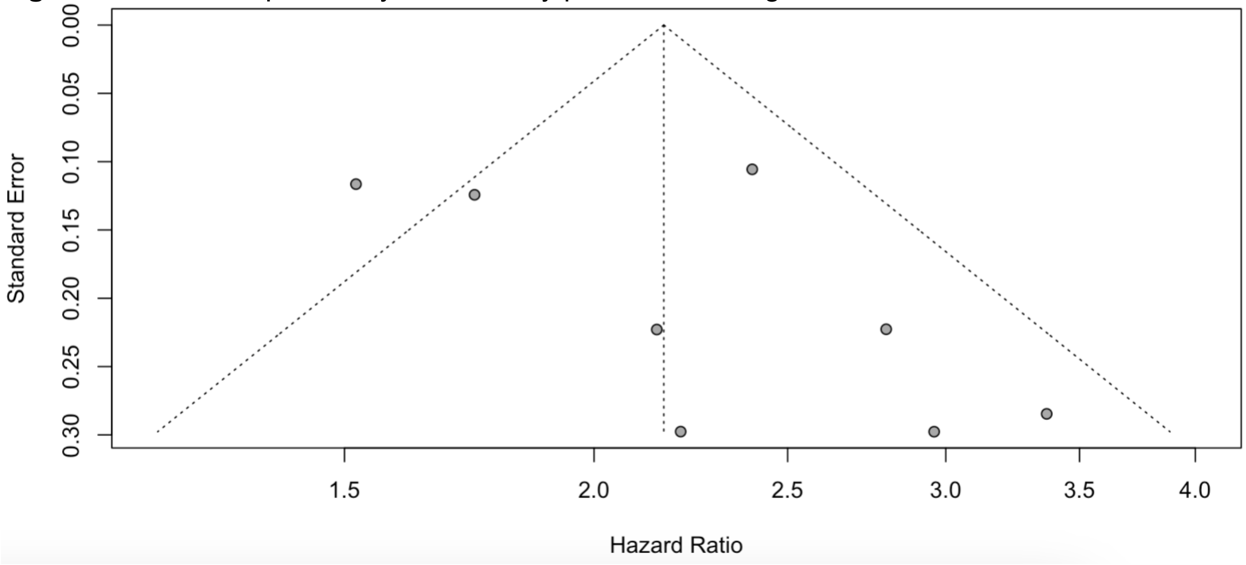

Figure S13. Funnel plot of 3-year mortality pooled HR using random-effect model

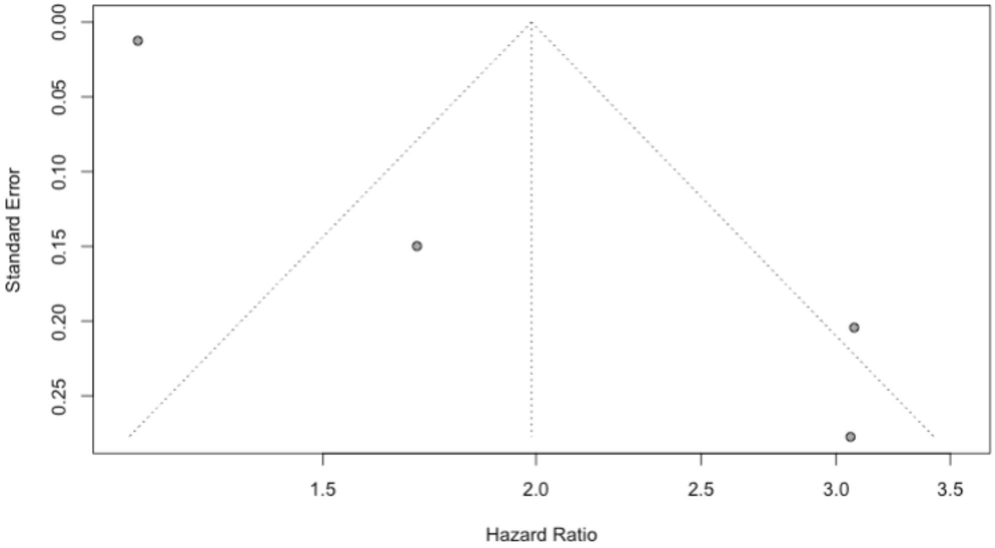

PRISMA 2020 CHECKLIST

Table S6. PRISMA 2020 Checklist

Section and Topic	Item #	Checklist item	Location where item is reported
**TITLE**			
Title	1	Identify the report as a systematic review.	Title page
**ABSTRACT**			
Abstract	2	See the PRISMA 2020 for Abstracts checklist.	Abstract
**INTRODUCTION**			
Rationale	3	Describe the rationale for the review in the context of existing knowledge.	line 40-64
Objectives	4	Provide an explicit statement of the objective(s) or question(s) the review addresses.	line 65-69
**METHODS**			
Eligibility criteria	5	Specify the inclusion and exclusion criteria for the review and how studies were grouped for the syntheses.	line 76-86
Information sources	6	Specify all databases, registers, websites, organisations, reference lists and other sources searched or consulted to identify studies. Specify the date when each source was last searched or consulted.	line 88-91
Search strategy	7	Present the full search strategies for all databases, registers and websites, including any filters and limits used.	line 93-94; Table 1
Selection process	8	Specify the methods used to decide whether a study met the inclusion criteria of the review, including how many reviewers screened each record and each report retrieved, whether they worked independently, and if applicable, details of automation tools used in the process.	line 89-97
Data collection process	9	Specify the methods used to collect data from reports, including how many reviewers collected data from each report, whether they worked independently, any processes for obtaining or confirming data from study investigators, and if applicable, details of automation tools used in the process.	line 95-97
Data items	10a	List and define all outcomes for which data were sought. Specify whether all results that were compatible with each outcome domain in each study were sought (e.g. for all measures, time points, analyses), and if not, the methods used to decide which results to collect.	line 99-103; line 140-145
	10b	List and define all other variables for which data were sought (e.g. participant and intervention characteristics, funding sources). Describe any assumptions made about any missing or unclear information.	Line 101-103; line 159-162
Study risk of bias assessment	11	Specify the methods used to assess risk of bias in the included studies, including details of the tool(s) used, how many reviewers assessed each study and whether they worked independently, and if applicable, details of automation tools used in the process.	line 105-108
Effect measures	12	Specify for each outcome the effect measure(s) (e.g. risk ratio, mean difference) used in the synthesis or presentation of results.	line 110-122
Synthesis methods	13a	Describe the processes used to decide which studies were eligible for each synthesis (e.g. tabulating the study intervention characteristics and comparing against the planned groups for each synthesis (item #5)).	line 115-132
	13b	Describe any methods required to prepare the data for presentation or synthesis, such as handling of missing summary statistics, or data	line 115-132;

Section and Topic	Item #	Checklist item	Location where item is reported
		conversions.	line 119-121; line 159-162
	13c	Describe any methods used to tabulate or visually display results of individual studies and syntheses.	line 115-132
	13d	Describe any methods used to synthesize results and provide a rationale for the choice(s). If meta-analysis was performed, describe the model(s), method(s) to identify the presence and extent of statistical heterogeneity, and software package(s) used.	line 115-173
	13e	Describe any methods used to explore possible causes of heterogeneity among study results (e.g. subgroup analysis, meta-regression).	line 136-138; line 170-171
	13f	Describe any sensitivity analyses conducted to assess robustness of the synthesized results.	line 164-169
Reporting bias assessment	14	Describe any methods used to assess risk of bias due to missing results in a synthesis (arising from reporting biases).	line 171-173
Certainty assessment	15	Describe any methods used to assess certainty (or confidence) in the body of evidence for an outcome.	Not Applicable
**RESULTS**			
Study selection	16a	Describe the results of the search and selection process, from the number of records identified in the search to the number of studies included in the review, ideally using a flow diagram.	line 175-186, Figure 1
	16b	Cite studies that might appear to meet the inclusion criteria, but which were excluded, and explain why they were excluded.	Figure 1
Study characteristics	17	Cite each included study and present its characteristics.	Table 2
Risk of bias in studies	18	Present assessments of risk of bias for each included study.	Supplementary Table 1
Results of individual studies	19	For all outcomes, present, for each study: (a) summary statistics for each group (where appropriate) and (b) an effect estimate and its precision (e.g. confidence/credible interval), ideally using structured tables or plots.	Figure 2, Figure 3
Results of syntheses	20a	For each synthesis, briefly summarise the characteristics and risk of bias among contributing studies.	Table 2, Supplementary Table 1
	20b	Present results of all statistical syntheses conducted. If meta-analysis was done, present for each the summary estimate and its precision (e.g. confidence/credible interval) and measures of statistical heterogeneity. If comparing groups, describe the direction of the effect.	line 201-260, Figure 2-4, Supplementary Table 2-4 & 6, Supplementary Figure 8
	20c	Present results of all investigations of possible causes of heterogeneity among study results.	line 201-260
	20d	Present results of all sensitivity analyses conducted to assess the robustness of the synthesized results.	line 248-255
Reporting biases	21	Present assessments of risk of bias due to missing results (arising from reporting biases) for each synthesis assessed.	line 257-260

Section and Topic	Item #	Checklist item	Location where item is reported
			Supplementary Figure 9-13
Certainty of evidence	22	Present assessments of certainty (or confidence) in the body of evidence for each outcome assessed.	Not Applicable
**DISCUSSION**			
Discussion	23a	Provide a general interpretation of the results in the context of other evidence.	line 269-310
	23b	Discuss any limitations of the evidence included in the review.	line 312-327
	23c	Discuss any limitations of the review processes used.	line 312-327
	23d	Discuss implications of the results for practice, policy, and future research.	line 296-297, line 301-302, line 308-310, line 339-341
**OTHER INFORMATION**			
Registration and protocol	24a	Provide registration information for the review, including register name and registration number, or state that the review was not registered.	line 70-74
	24b	Indicate where the review protocol can be accessed, or state that a protocol was not prepared.	line 70-74
	24c	Describe and explain any amendments to information provided at registration or in the protocol.	Not Applicable
Support	25	Describe sources of financial or non-financial support for the review, and the role of the funders or sponsors in the review.	line 356
Competing interests	26	Declare any competing interests of review authors.	line 356
Availability of data, code and other materials	27	Report which of the following are publicly available and where they can be found: template data collection forms; data extracted from included studies; data used for all analyses; analytic code; any other materials used in the review.	line 353, line 358 Supplementary materials
